# The Most Frequently Observed Oto-Laryngeal Affections and Wounds

**Published:** 1918-09

**Authors:** A. Hautant


**The Most Frequently Observed Oto-Laryngeal Affections and Wounds, and the Means of Treatment to Avoid Wastage.** By Médecin-Major A. Hautant.

The speaker said in part :

*Chronic suppuration of the middle ear*, complicates more than half of the cases. The majority of these date from before the war, and others are due to acute traumatic otitis. It would have been possible by treatment with glycerine, alcohol, and tincture of iodine in equal parts to avoid a great number of thecases of chronic otorrhea which have come under our observation.

1. Apart from the cases of suspected tuberculosis of the ear, and serious lesion, all other causes of chronic otorrhea are compatible with active service. If a case of otorrhea is healthy and robust, why eliminate him from fighting units?

2. During the war, the treatment of otorrhea must be palliative and conservative. One must be very chary of surgical intervention, and take into consideration the septic condition of the operative area, and also the lack of good will on the part of the patient.

Thus, after two years the speaker decided that conservative treatment was the best for cases of otorrhea, destruction of the ear-drum, distribution of the granulation, and ablation of polypi.

As a sort of corollary to this medical routine, there is a corresponding military routine. The regimental doctor must be informed as to the nature of the man's affection and the treatment that the patient himself is capable of bringing to bear.

*Deafness through Nervous Commotion*

This may be caused simply by shell-burst without any physical lesion. During the first two years of the war, these cases were very frequent. The last two years they have been less so. This diminution may have been due to the fact that the men later became accustomed to loud explosions. Instead of leaving these patients in the ordinary hospitals, they are gathered together and treated immediately.

1. *War deafness* caused by simple commotion is a reality, but in general it is but transitory.

2. In certain cases, however war deafness remains.

The question arises as to whether the patient is a malingerer, or whether he is subjet to true nervous deafness. Apart from the instances of simulation, in the majority of cases of war deafness the patients are genuine, and their auditory disturbances may arise from two sources:

1. Labyrinthine.
2. Endocranian.

That the latter is frequent is revealed by the resut of lumbarl puncture.

On the whole, war deafness seems to be dependent upon fairly superficial anatomical alterations, and, if it becomes marked, it is because of the introduction of the psychic element. Definite war deafness can be avoided by combating that psychic element.

There are two means of doing this.

1. Initial treatment.
2. Auditory re-education.

It might be possible to forestall war deafness because it is nearly always superadded to an auricular condition which is often transitory. Or it may even be supperadded to a former auricular condition. It might not be a bad plan to place all men with these old auricular lesions in the secondary services, thereby not exposing them to violent bombardments

### *Aphonia.*

*Etiology.* There are two main causes.

*a*) The after-consequences of ordinary acute laryngitis.

*b*) Gas intoxication.

II. *Lesion.*

These are diverse in character.

*a*) In the first group are the causes of simple muscular weakness — in other words, cases of asthenic laryngitis.

*b*) In the second group are lesions of chronic hypertrophic laryngitis.

These lesions are often diffuse, although they are also often confined to the inter-arytenoidal space.

Frequently tuberculous laryngitis consequent upon a phase of non-ulcerated infiltration is suspected — an error which cannot be too strongly emphasized.

III. *Treatment.*

*a*) Prophylactic, which consists of the cleaning out and the disinfection of the nasal passages.

*b*) Curative, which consists of respiratory and vocal re-education.

In short : —

Apart from serious surgical affections of the ear, nose, and throat, practically every case of oto-laryngitis may return to his fighting unit.

The frequency of these infections may be greatly diminished by keeping the nasal passage and the naso-pharyngeal cavity in good condition.

In confirmed cases simple palliative methods may be used, but very seldom surgical intervention. Even if these cases prove obstinate, the medical officer must not lose patience. He will often bring about sufficient improvement to admit of the man's return to the front.

